# Pain Acceptance and Pain-Related Disability Predict Healthcare Utilization and Medication Intake in Patients with Non-Specific Chronic Spinal Pain

**DOI:** 10.3390/ijerph17155556

**Published:** 2020-07-31

**Authors:** Rosa Esteve, Alicia Eva López-Martínez, Gema Teresa Ruíz-Párraga, Elena Rocío Serrano-Ibáñez, Carmen Ramírez-Maestre

**Affiliations:** 1 Instituto de Investigación Biomédica de Málaga, Facultad de Psicología, Andalucía Tech, Universidad de Málaga, 29071 Málaga, Spain; zarazaga@uma.es (R.E.); aelm@uma.es (A.E.L.-M.); gtruizparraga@uma.es (G.T.R.-P.); elenarserrano@uma.es (E.R.S.-I.); 2 Facultad de Ciencias de la Salud, Área de Psicología, Universidad Isabel I, 09003 Burgos, Spain

**Keywords:** pain acceptance, chronic pain, disability, healthcare utilization, medication intake, prospective study

## Abstract

Longitudinal research is needed to determine predictive factors of healthcare utilization and medication intake in people with non-specific spinal pain. This study aims to prospectively examine the predictive value of sex, age, work status, pain intensity, pain acceptance, disability, depression, pain anxiety, and catastrophizing in relation to healthcare utilization and medication intake in people with non-specific spinal pain. Participants were 79 patients with non-specific spinal pain of 6 to 9 months’ duration. They were followed-up at 6 months and 12 months. At enrolment they were administered a battery of questionnaires assessing the predictive variables. Healthcare utilization and medication intake were assessed at follow-ups 1 and 2. At follow-up 1, higher pain acceptance was associated with less healthcare utilization and less medication intake, while male sex was associated with less medication intake. At follow-up 2, higher pain-related disability was associated with higher healthcare use, and pain intensity was associated with higher medication intake. These results suggest that during the early stages of non-specific spinal pain chronification, pain acceptance and the avoidance of pain-related disability—understood as giving up normal activities—can lead to reductions in healthcare utilization and medication intake.

## 1. Introduction

Approximately one billion adults worldwide experience non-specific spinal pain [1]. The term “non-specific pain” is used because this type of pain cannot be reliably attributed to a specific underlying condition, such as cancer, infection, or inflammatory disease [2]. Due to this absence of underlying disease, healthcare professionals find the clinical assessment and treatment of such patients extremely difficult [3]. The use of healthcare services and medication intake is not only higher in patients with chronic pain than in the general population, but is even higher in those with spinal pain [4]. Thus, health service funders and patients experience increased costs [5]. Apart from the economic aspects, there is a risk of over-treatment. Furthermore, patients often experience high levels of emotional frustration because of the apparently endless succession of fruitless tests, consultations, and changes in medication. These feelings of frustration are often associated with both anger and a sense of perceived injustice, which are typically directed toward health professionals [6,7,8]. Thus, the rehabilitation process is hindered [9,10].

A recent review showed that very few studies have analyzed the association between the increased use of healthcare services and chronic pain, despite the recognized importance of this topic [11]. Associations have been found between the increased use of healthcare services and factors, such as pain-related disability [12], pain intensity [13], age [14], depression [15], and sex [16]. A Spanish study found that the healthcare system was mainly used by individuals who leave or lose their job as a result of pain [17]. Associations have been found between some psychological factors related with pain chronification (e.g., fear-avoidance beliefs and pain catastrophizing) and healthcare service use [18,19].

Evidence has also been found of associations between higher pain acceptance and fewer medical visits and less drug consumption [20,21,22,23]. Acceptance is a key process within the Psychological Flexibility Model [24]. Pain acceptance entails responding to pain-related experiences without attempts at control or avoidance [20]. This aspect is of particular relevance when such attempts limit the patients’ quality of life and stop them from engaging in valued activities and reaching personal goals [25]. From this theoretical perspective, high medication consumption and treatment seeking, in contrast to an acceptance response, have been conceptualized as forms of avoidance behavior [25]. Several prospective cohort studies have found an association between pain acceptance and better general functioning and work status, and less disability, emotional distress, and healthcare utilization in groups of patients with chronic pain, regardless of their pain intensity [20,21,22].

Thus, it is relevant to (a) study the predictive power of pain acceptance in relation to healthcare utilization and medication intake, and (b) investigate the status of this construct as a meaningful addition to other predictive factors that have been the object of previous studies. Cross-sectional studies are the main source of results regarding these factors, and thus, longitudinal research is needed to determine their predictive power. The study objective was to prospectively assess the predictive value of pain acceptance and the aforementioned factors in relation to healthcare utilization and medication intake in patients with non-specific spinal pain. The following hypotheses were formulated:

**Hypothesis 1** **(H1).** Increased healthcare service use and medication intake would be prospectively predicted by pain-related disability, pain intensity, depression, age, sex, work status, pain anxiety, and pain catastrophizing.

**Hypothesis 2** **(H2).** Reduced healthcare service use and medication intake would be prospectively predicted by pain acceptance.

To the best of our knowledge, this is the first prospective study to investigate the predictive value of pain acceptance and a wide range of other factors that have been investigated in other studies. In contrast to such studies [20,21,22], all the participants recruited in the present study were (a) in the early phase of chronic non-specific spinal pain, (b) had been experiencing pain for a similar period (i.e., 6–9 months), and (c) were followed up over a long period (1 year), which included two measurement occasions.

## 2. Materials and Methods 

### 2.1. Procedure

This study was part of a larger research project [26]. The project was conducted in accordance with the Declaration of Helsinki and received ethical clearance by the Institutional Ethics Review Board (ERC UMA) and the Regional Hospital Ethics Committee. At the end of visits to their doctors, the patients who met the eligibility criteria were informed of the study aims, and their participation was requested. Some participants were interviewed after their visit, whereas others left their telephone number to make an appointment on another day. Written informed consent was obtained prior to data collection. Each participant had a semi-structured interview with a psychologist and also completed a battery of questionnaires. The approximate duration of the session was 45 minutes. Baseline demographic and clinical variables were assessed, as well as the predictive variables. Healthcare utilization and medication intake related to the primary pain complaint were assessed at follow-ups 1 and 2. Five primary care units participated in the study, which were located in different urban and rural areas. The participants were always interviewed in their usual primary care center. 

### 2.2. Participants

General practitioners in five primary care units recruited 86 patients whose primary complaint was non-specific spinal pain. Individuals were considered eligible for inclusion if they met the following criteria: (a) being between 18 and 65 years of age; (b) able to understand written and spoken Spanish; (c) had non-specific spinal pain in the cervical, thoracic, or lumbosacral regions; (d) spinal pain of 6 to 9 months’ duration; and (e) pain intensity equal to or higher than 3 on a 10-point scale.

The exclusion criteria were: (a) being treated for malignant tumors, terminal illness, or psychiatric disorder; (b) spinal pain that was related to or secondary to a specific medical condition (tumors, trauma, infection, fractures, and inflammatory disorders); (c) chronic widespread syndromes (e.g., fibromyalgia, chronic fatigue syndrome); (d) surgery in the spinal area; and (e) pregnancy. Seven of the participants (9%) were excluded because they did not meet the inclusion criteria, or they met one of the exclusion criteria.

In total, 79 patients participated in the study (64 women and 15 men). All participants were Caucasian, and their average age was 50.89 years (SD = 14.11). The marital status of the participants was as follows: married (59.50%), never married (27.80%), cohabiting (3.8%), widowed (5.1%), and separated (3.8%). In total, 38% had completed primary school, 34.2% had completed high school, and 16.5% had a university degree. Pain sites were as follows: cervical (59.5%), vertebral-lumbar (32.9%), sacral (30.4%), thoracic (30.4%), and lumbar-renal (21.5%).

**Figure 1:**
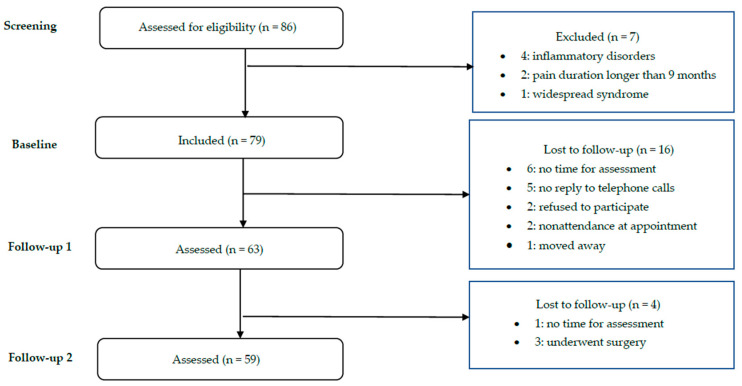
Flow chart of inclusion.

The participants were assessed on three occasions (see Figure 1 for the study flowchart). The baseline assessment was conducted at 6 to 9 months of pain duration and two more assessments were made at 6-month intervals. The two follow-ups (i.e., follow-ups 1 and 2) were specifically scheduled independently from the participants’ standard medical checkups. Sixty-three participants completed follow-up 1, and 59 participants completed follow-up 2. The participants who completed the three assessment sessions (*N* = 59) were compared to those who did not complete them. No significant differences were found between the groups in relation to any of the study variables.

### 2.3. Variables and Instruments

#### 2.3.1. Demographic and Clinical Pain-Related Variables

A semi-structured interview was used to collect data on demographic and pain-related variables including age, sex, civil status, education, work status, pain duration, pain location, medications being taken, and healthcare use. 

#### 2.3.2. Pain intensity

Participants were asked to rate their least, average, and worst pain during the previous two weeks, as well as their current pain, on a scale ranging from 0 to 10. A rating of 0 indicated “no pain”, and 10 indicated pain “as intense as you could imagine”. A composite pain intensity score was calculated for each participant by calculating the average of the least, average, worst, and current pain, with higher scores indicating higher perceived pain intensity. Composites of the 0–10 ratings are very reliable measures of pain intensity in patients with chronic pain [27]. 

#### 2.3.3. Anxiety and Depression Scale

Depression was assessed using the Depression subscale of the Spanish version of the Hospital Anxiety and Depression Scale, which is a self-reporting scale comprising seven items (item score 1–4) [28,29]. The total score ranges from 0 to 28, with higher scores indicating higher depression. The internal consistency of the subscale is high (α = 0.86) [28].

#### 2.3.4. Pain Catastrophizing 

Pain-related catastrophizing was assessed using the Spanish adaptation of the Pain Catastrophizing Scale [30,31]. It consists of three subscales assessing rumination, magnification and helplessness and also provides a total score on catastrophizing. The total score alone was used in this study. Items are scored on a five-point ordinal scale. The total score ranges from 0 to 52 points, with higher scores corresponding to higher levels of pain catastrophizing. The Spanish version of the scale shows appropriate reliability and validity. Internal consistency was high (α = 0.79) [30]. 

#### 2.3.5. Pain Anxiety

Anxiety and fear responses associated with chronic pain were measured using the Spanish adaptation of the Pain Anxiety Symptoms Scale [32,33]. This scale comprises 20 items scored on a six-point scale. The total score ranges from 0 to 100 points, with higher scores indicating higher levels of pain anxiety. The Spanish version of the questionnaire showed high internal consistency (α = 0.93) [32]. 

#### 2.3.6. Pain Acceptance

Pain acceptance was measured using the Spanish version of the Pain Acceptance Questionnaire [34,35]. This instrument comprises 20 items (item score 0–6). The total score ranges from 0 to 120, with higher scores indicating higher levels of pain acceptance. Similar to the original questionnaire, the Spanish version yields a total score and two subscale scores for pain willingness and activity engagement. In addition, the CPAQ-SV demonstrates good criterion validity [34]. The total score alone was used in this study and showed appropriate internal consistency (α = 0.83).

#### 2.3.7. Impairment and Functioning 

Pain-related disability was assessed using the Impairment index of the Impairment and Functioning Inventory [36]. This 30-item instrument assesses the number of activities that patients have given up since pain began. The total score ranges from 0 to 30, with higher scores indicating higher pain-related disability. The impairment subscale shows good internal consistency (α = 0.98) and good criterion validity [36].

#### 2.3.8. Healthcare Utilization

To assess healthcare utilization, respondents were asked questions on medical care sought for any spinal pain they had experienced since the previous interview. These questions specifically addressed the following aspects: (1) the number of general practitioner consultations; (2) the number of medical specialist consultations; (3) the number of physical therapist visits; and (4) the number of hospital emergency attendances. This study defined the outcome of interest as the total number of contacts with healthcare services. It has been found that data on healthcare utilization collected using self-reports and data collected from medical records are of comparable quality [37]. 

#### 2.3.9. Medication Intake

Participants were also asked about the number of different medications they were taking. The variable “medication intake” was defined as the sum of the different categories of pain medications being taken at the time of assessment: weak opioids, strong opioids, non-steroid anti-inflammatory drugs, tricyclic antidepressants, muscle relaxants, sedatives, anticonvulsants, selective serotonin reuptake inhibitors, and over-the-counter analgesics. 

### 2.4. Statistical Analysis

Data analyses were performed using the Statistical Package for the Social Sciences version 22.0 (SPSS; Chicago, IL, USA). Firstly, categorical variables were expressed as frequencies, and continuous variables were expressed as means and standard deviations. Secondly, regression analyses were performed. Healthcare utilization and medication intake (number of medications consumed) are count response data. The Poisson regression model is the standard method used to model such data; however, its application is typically limited by the requirement of equality of means and variances. In the present study, the variances of the variables healthcare utilization and medication intake were greater than their means. This result was mainly due to the high frequency of zero data: that is, the data were over-dispersed. Thus, due to the skewed data distribution, negative binomial regression analyses were conducted. The regression coefficients provided are not directly interpretable because the negative binomial distribution was non-linear. Therefore, the coefficients were transformed into incident rate ratios (IRRs) by computing their exponentials (i.e., eβ). Firstly, all the proposed explanatory variables were screened for univariate associations with the dependent variables. Secondly, the explanatory variables that had a significant association with the dependent variables were included in the multivariate analysis. The probability of making a Type I error increases when conducting multiple analyses on the same outcome variable. Thus, the Bonferroni correction using a p value of 0.005 as a threshold to control Type I errors was applied. Negative binomial regression has been found to be robust with moderate sample sizes (>50) [38]. 

## 3. Results

Table 1 shows the frequencies for categorical variables and means and standard deviations for continuous variables, and Table 2 shows the comparison of women and men on the predictor and outcome variables. 

Regarding healthcare utilization, at follow-up 1, univariate negative binomial regression analysis (Table 3) showed that higher pain acceptance was associated with less healthcare utilization (IRR = 0.984; CI: 0.973–0.995, *p* = 0.004): that is, each one-point increase in pain acceptance scores was associated with a 1.6% decrease in healthcare use. At follow-up 2 (i.e., 12 months after the baseline assessment), the only significant association was between pain-related disability and healthcare utilization (IRR = 1.071; CI: 1.033–1.111, *p* = 0.000): that is, each one-point increase in pain-related disability scores was associated with a 7.1% increase in healthcare use. 

Regarding medication intake, at follow-up 1 (i.e., 6 months after the initial assessment), univariate negative binomial regression analysis (Table 4) showed a significant association between medication intake and male sex (IRR = 0.341; CI: 0.163–0.713, *p* = 0.004), pain intensity (IRR = 1.191, CI: 1.056–1.344, *p* = 0.004), pain anxiety (IRR = 1.016, CI: 1.007–1.024, *p* = 0.000), and pain acceptance (IRR = 0.979, CI: 0.969–0.990, *p* = 0.000). A multivariate contrast that introduced these four variables showed that only male sex (IRR = 0.311, CI: 0.149–0.649, *p* = 0.002) and pain acceptance (IRR = 0.978, CI: 0.968–0.989, *p* = 0.000) were significantly associated with medication intake. Male sex was associated with a 68.9% decrease in medication intake. In addition, each one-point increase in pain acceptance scores was associated with a 2.2% decrease in medication intake. At follow-up 2, univariate negative binomial regression analysis only showed a significant association between pain intensity and higher medication intake (IRR = 1.148, CI: 1.044–1.263, *p* = 0.004): that is, each one-point increase in pain intensity scores was associated with a 14.8% increase in medication intake. 

## 4. Discussion

The present study’s objective was to prospectively assess the predictive value of pain acceptance and pain-related disability, pain intensity, depression, age, sex, work status, pain anxiety, and pain catastrophizing in relation to healthcare utilization and medication intake in people with non-specific spinal pain. Negative binomial regression analyses showed that pain acceptance significantly predicted healthcare utilization and medication intake when participants had experienced non-specific spinal pain for 12 to 15 months (follow-up 1). Six months later, pain-related disability and pain intensity predicted healthcare utilization and medication intake, respectively (follow-up 2). At follow-up 1, male sex significantly predicted lower medication intake. Contrary to the hypotheses, the rest of the variables did not significantly predict neither healthcare utilization nor medication intake. 

The results of the present study showed that an acceptance response was associated with less healthcare utilization and medication intake. This finding is in line with the results of previous and prospective studies [20,21,22], which have shown that pain acceptance is predictive of healthcare utilization and medication intake. Comparisons with the results of these studies must take into account the following aspects: the present study had a follow-up period of 1 year and included two measurement occasions apart from that conducted at baseline. In contrast, the aforementioned studies had follow-up periods of 3.9 months, 3.7 months, and 4.6 months, respectively, and included just one measurement occasion. “Doctor shopping” and high medication intake characterize what has been called an acute pain response [39]. In contrast to an acceptance response, individuals with an acute pain response seek a total cure for their pain and put life goals on hold. Healthcare professionals should be ready to recognize this response in their patients. Several studies have shown that when total remission is the only goal contemplated by patients with chronic pain, there is a mismatch between the patients’ and providers’ objectives [40,41] and patients will not be able to appreciate their progress. In this respect, a recent study in a sample of patients with spine pain showed that physiotherapeutic treatment was perceived as more effective among those with high acceptance than among those who reported low acceptance [42]. Goals set by the clinician and the patients are crucial to promoting adherence during the rehabilitation process [43,44,45]. This therapeutic strategy can be hindered by differences between the clinicians and patients concerning such goals. Healthcare professionals should encourage an acceptance response. If needed, they should refer patients to a psychologist specializing in cognitive behavioral therapy [46] or Acceptance and Commitment therapy, as these approaches have proven to be effective in reducing healthcare utilization and medication intake [47]. 

At follow-up 2, pain-related disability and pain intensity predicted healthcare utilization and medication intake, respectively. These results are consistent with a disability response. Patients with this response assume that pain is likely to be chronic. They see themselves as disabled, are highly sedentary, have abandoned their life goals, and are often depressed. There is increasing evidence that disability levels, rather than pain intensity, are the main reason patients with non-specific low back pain consult healthcare providers [12]. This shift in the predictive variables could be understood as a process-of-change model of pain self-management [48,49,50]. Based on this perspective and our results, it seems that during the first months of the chronic pain experience an acceptance response could prevent patients from using a pain management strategy that is exclusively centered on medical solutions. However, over time, their level of disability would be the determining factor in their approach to pain management. Thus, there is a need to (a) clearly identify the activities that the patients have abandoned since they began to experience spinal pain, and (b) encourage an acceptance response at an early stage of pain chronification given that pain acceptance entails engaging in normal life activities even if pain is present [20]. In this regard, international guidelines for the management of non-specific low back pain recommend an early return to normal activity and exercise [51]. 

At follow-up 2, pain intensity predicted medication intake. This result was similar to those of other prospective studies [52]. In addition, it has been suggested that (a) nociceptive processes are not only relevant in patients with acute pain, but also in patients with chronic pain, and that (b) the value of pain as a biological signal should not be underestimated [53]. Furthermore, the finding that sex was a predictor of medication use at the first measurement occasion could have been due to the application of different prescription patterns according to the patients’ sex. Recent reviews have found gender-related biases in pain treatment that are due to the interaction of several factors. These factors include the characteristics of both the patients and the care providers [54,55]. Specifically, female healthcare providers prescribed more antidepressants to female patients than to male patients, and sedatives were more frequently prescribed to men [54,55].

The present study has some limitations. Firstly, many efforts have been made to reduce the possibility of Type I errors; however, Type II errors may be present in our analyses and significant associations may remain undetected. Secondly, there may have been differences in the unmeasured variables between dropouts and participants who completed the study, even though the dropout rate was low, and no significant differences were found between the participants who completed the three assessment sessions and those who did not. In order to prevent participant dropout or to reduce the dropout rate, future research should compensate the patients economically for taking part in the study. However, we were unable to do this due to restrictions imposed at our university on the use of financial incentives in clinical research. Thirdly, self-reporting was the only method used, and thus shared method variance may have influenced the results. Moreover, inaccurate recall could have biased the information provided by the participants on healthcare utilization. Nevertheless, several studies have shown good levels of reliability and high congruency rates between self-reporting and medical records for recall periods of up to 12 months [56]. Thus, future research replicating the present study should also include different assessment methods. Fourthly, only a small amount of the total variance in healthcare utilization and medication intake was explained by the variables included in the present study. Thus, in order to construct a predictive model, future research should consider other theoretically relevant variables. In this regard, a recent study has highlighted the relevance of health-seeking behavior: patients who were high health-seekers before the initial consultation for spine pain had a two and a half to three times greater risk of being high healthcare utilizers after consultation for spine pain [57]. Additionally, the present study did not adjust for any covariates, and this is a limitation that should be addressed in future research with larger samples. Fifthly, the duration of the present study was 1.5 years (counting from the baseline assessment to the 2nd follow-up). Future research should include longer follow-up periods to obtain a more complete picture of how healthcare utilization and medication intake evolves in patients with non-specific back pain. Finally, it should be acknowledged that the use of longitudinal data cannot establish causality at the same level of certainty as that obtained using experimental methods.

## 5. Conclusions

In conclusion, besides pain intensity, psychological factors predict healthcare utilization and medication intake. Interestingly, the results of the present study suggest that the predictors changed over the course of time: at the beginning of the study, pain acceptance played a prominent role as a predictor, whereas 6 months later, disability—understood as the activities that patients have quit since they were in pain—became a predictor. Thus, during the early stages of spinal pain, an intervention program to encourage pain acceptance and prevent the abandonment of daily activities could contribute to reducing healthcare service costs and to improving the outcomes of rehabilitation interventions. The results of this study also indicated that medication prescriptions may be affected by sex bias. Future research should investigate how medical education could be improved, such that sex bias can be avoided when making prescriptions.

## Figures and Tables

**Table 1:** Means and percentages of the predictive and outcome variables.

Variable	Mean (SD)	*N* (%)
Sex		
Female		50 (79.4)
Male		13 (20.6)
Employment status		
Full-Time employed		29 (46)
Homemaker		21 (33.3)
Other (student, retired, unemployed)		13 (20.7)
Pain intensity	4.79 (2.16)	
Age	49.57 (16.49)	
Depression	11.54 (4.81)	
Pain catastrophizing	15.40 (12.50)	
Pain anxiety	57.29 (29.35)	
Pain acceptance	72.54 (22.26)	
Disability	2.71 (3.73)	
Healthcare utilization follow-up 1	1.76 (1.78)	
Healthcare utilization follow-up 2	0.85 (0.83)	
Medication intake follow-up 1	0.95 (0.91)	
Medication intake follow-up 2	1.20 (0.98)	

**Table 2:** Comparison of women and men on the predictor and outcome variables. T-tests and chi-square.

Variable	Women		Men		t/χ^2^	*p*
	Mean/*N*	SD	Mean/*N*	SD		
Employment status	Full-Time employed: 21 Homemaker: 21 Other (student, retired, unemployed): 8		Full-Time employed: 8 Homemaker: 0 Other (student, retired, unemployed): 5		8.838	0.012
Pain intensity	5.05	1.89	3.81	2.85	−1.487	0.158
Age	50.66	16.58	45.38	16.09	−1.028	0.308
Depression	11.64	4.62	11.15	5.68	−0.322	0.748
Pain Catastrophizing	14.74	12.06	17.92	14.29	0.816	0.418
Pain anxiety	58.14	29.70	54.00	28.91	−0.450	0.654
Pain acceptance	72.34	20.34	73.31	29.50	0.139	0.890
Disability	2.78	3.55	2.46	4.50	−0.272	0.786
Healthcare utilization follow-up 1	1.80	1.81	1.62	1.71	−0.332	0.741
Healthcare utilization follow-up 2	0.84	0.81	0.88	0.89	−0.354	0.725
Medication intake follow-up 1	1.10	0.91	0.38	0.65	−2.658	0.010
Medication intake follow-up 2	1.35	0.97	0.81	0.91	−1.783	0.080

**Table 3:** Univariate negative binomial regression analysis of healthcare use.

Predictors	Follow-Up 1			Follow-Up 2		
	IRR	95% CI	*p*	IRR	95% CI	*p*
Sex (female/male)	0.949	0.498–1.807	0.874	1.045	0.596–1.832	0.877
Employment status						
Full-time employed	0.757	0.381–1.502	0.425	0.861	0.476–1.558	0.622
Homemaker	0.673	0.323–1.401	0.290	0.805	0.424–1.529	0.507
Other	1	----	----	1	----	----
Pain intensity	1.154	1.020–1.304	0.023	1.019	0.909–1.143	0.744
Age	1.001	0.985–1.017	0.935	0.989	0.975–1.004	0.164
Depression	1.013	0.961–1.068	0.635	1.020	0.972–1.070	0.415
Pain catastrophizing	1.010	0.989–1.031	0.362	1.009	0.991–1.028	0.339
Pain anxiety	1.009	1.000–1.018	0.041	1.007	0.998–1.016	0.113
Pain acceptance	0.984	0.973–0.995	0.004	0.993	0.983–1.004	0.200
Disability	1.072	1.004–1.145	0.037	1.071	1.033–1.111	0.000

*Note.* IRR: Incidence Rate Ratio; CI: Confidence Interval. Bonferroni adjusted *p*-values.

**Table 4:** Univariate negative binomial regression analysis of medication intake.

Predictors	Follow-Up 1			Follow-Up 2		
	IRR	95% CI	*p*	IRR	95% CI	*p*
Sex (female/male)	0.341	0.163–0.713	0.004	0.602	0.364–0.998	0.049
Employment status						
Full-time employed	1.144	0.554–2.362	0.717	0.909	0.541–1.529	0.719
Homemaker	2.519	1.231–5.154	0.011	1.444	0.853–2.445	0.172
Other	1	---	---	1	---	---
Pain intensity	1.191	1.056–1.344	0.004	1.148	1.044–1.263	0.004
Age	1.017	1.001–1.034	0.041	1.011	0.997–1.025	0.121
Depression	1.061	1.014–1.111	0.011	1.053	1.013–1.094	0.008
Pain catastrophizing	1.024	1.005–1.043	0.014	1.006	0.990–1.022	0.440
Pain anxiety	1.016	1.007–1.024	0.000	1.008	1.001–1.016	0.031
Pain acceptance	0.979	0.969–0.990	0.000	0.988	0.980–0.997	0.011
Disability	1.036	0.972–1.104	0.277	1.014	0.957–1.075	0.637

Note. IRR: Incidence Rate Ratio; CI: Confidence Interval. Bonferroni adjusted *p*-values.

## References

[1] Global Burden of Disease 2013 Collaborators (2013). Global, Regional, and National Incidence, Prevalence, and Years Lived with Disability for 301 Acute and Chronic Diseases and Injuries in 188 Countries, 1990–2013: A Systematic Analysis for the Global Burden of Disease Study 2013. Lancet.

[2] Treede R.D., Rief W., Barke A., Aziz Q., Bennett M.I., Benoliel R., Cohen M., Evers S., Finneruph N.B., First M.B. (2019). Chronic Pain as a Symptom or a Disease: The IASP Classification of Chronic Pain for the International Classification of Diseases: (ICD-11). Pain.

[3] Maher C.G., Underwood M., Buchbinder R. (2017). Non-Specific Low Back Pain. Lancet.

[4] Kim L.H., Vail D., Azad T.D., Bentley J.P., Zhang Y., Ho A.L., Fatemi P., Feng A., Varshneya K., Desai M. (2019). Expenditures and Health Care Utilization among Adults with Newly Diagnosed Low Back and Lower Extremity Pain. JAMA.

[5] The Pain Proposal 2010. http://www.efic.org/index.asp?sub=B57HFCF6J4043I..

[6] Bissell A.D., Ziadni M.S., Sturgeon A.J. (2018). Perceived Injustice in Chronic Pain: An Examination Through the Lens of Predictive Processing. Pain Manag..

[7] Scott W., Trost Z., Bernier E., Sullivan M.J. (2013). Anger Differentially Mediates the Relationship between Perceived Injustice and Chronic Pain Outcomes. Pain.

[8] Sullivan M.J., Scott W., Trost Z. (2012). Perceived Injustice. Clin. J. Pain.

[9] Carriere J.S., Sturgeon J.A., Yakobov E., Kao M.C., Mackey S.C., Darnall B.D. (2018). The Impact of Perceived Injustice on Pain-related Outcomes: A Combined Model Examining the Mediating Roles of Pain Acceptance and Anger in a Chronic Pain Sample. Clin. J. Pain.

[10] Sullivan M.J., Yakobov E., Scott W., Tait R. (2014). Perceived Injustice and Adverse Recovery Outcomes. Psychol. Inj. Law.

[11] Dueñas M., Ojeda B., Salazar-Couso A., Mico J.A., Failde I. (2016). A review of chronic pain impact on patients, their social environment and the health care system. J. Pain Res..

[12] Ferreira M., Machado G., Latimer J., Maher C., Ferreira P.H., Smeets R.J. (2010). Factors Defining Care-Seeking in Low Back Pain–A Meta-Analysis of Population Based Surveys. Eur. J. Pain.

[13] Tiira A., Paananen M., Taimela S., Zitting P., Järvelin M., Karppinen J.I. (2012). Determinants of Adolescent Health Care Use for Low Back Pain. Eur. J. Pain.

[14] Hirsch O., Strauch K., Held H., Redaelli M., Chenot J.-F., Leonhardt C., Keller S., Baum E., Pfingsten M., Hildebrandt J. (2014). Low Back Pain Patient Subgroups in Primary Care. Clin. J. Pain.

[15] Rayner L., Hotopf M., Petkova H., Matcham F., Simpson A., McCracken L.M. (2016). Depression in Patients with Chronic Pain Attending a Specialised Pain Treatment Center: Prevalence and Impact on Health Care Costs. Pain.

[16] Eriksen J., Sjøgren P., Ekholm O., Rasmussen N.K. (2004). Health Care Utilisation among Individuals Reporting Long-Term Pain: An Epidemiological Study Based on Danish National Health Surveys. Eur. J. Pain.

[17] Failde I. (2014). El Dolor Crónico, Algo Más Que Un Problema De Quien Lo Padece. Rev.Soc. Esp. Dolor.

[18] Jöud A., Björk J., Gerdle B., Grimby-Ekman A., Larsson B. (2017). The Association between Pain Characteristics, Pain Catastrophizing and Health Care Use – Baseline Results from the SWEPAIN Cohort. Scand. J. Pain.

[19] Keeley P., Creed F., Tomenson B., Todd C., Borglin G., Dickens C. (2008). Psychosocial Predictors of Health-Related Quality of Life and Health Service Utilisation in People with Chronic Low Back Pain. Pain.

[20] McCracken L.M., Eccleston C. (2005). A Prospective Study of Acceptance of Pain and Patient Functioning with Chronic Pain. Pain.

[21] McCracken L.M., Vowles K.E. (2008). A Prospective Analysis of Acceptance of Pain and Values-Based Action in Patients with Chronic Pain. Heal. Psychol..

[22] McCracken L.M., Vowles K.E., Gauntlett-Gilbert J. (2007). A Prospective Investigation of Acceptance and Control-Oriented Coping with Chronic Pain. J. Behav. Med..

[23] McCracken L.M., E Vowles K., Zhao-O’Brien J. (2010). Further Development of an Instrument to Assess Psychological Flexibility in People with Chronic Pain. J. Behav. Med..

[24] Hayes S.C., Luoma J.B., Bond F.W., Masuda A., Lillis J. (2006). Acceptance and Commitment Therapy: Model, Processes and Outcomes. Behav. Res. Ther..

[25] McCracken L.M., Morley S. (2014). The Psychological Flexibility Model: A Basis for Integration and Progress in Psychological Approaches to Chronic Pain Management. J. Pain.

[26] Esteve R., Bendayan R., López-Martínez A.E., Ramírez-Maestre C. (2017). Resilience and Vulnerability Factors When Pain is Acute as Predictors of Disability: Findings from a Two-Year Longitudinal Study. Pain Med.

[27] Jensen M.P., A Turner J., Romano J.M., Fisher L.D. (1999). Comparative Reliability and Validity of Chronic Pain Intensity Measures. Pain.

[28] Quintana J.M., Padierna A., Esteban C., Arostegui I., Bilbao A., Ruiz I. (2003). Evaluation of the psychometric characteristics of the Spanish version of the Hospital Anxiety and Depression Scale. Acta Psychiatr. Scand..

[29] Zigmond A.S., Snaith R.P. (1983). The Hospital Anxiety and Depression Scale. Acta Psychiatr. Scand..

[30] García-Campayo J., Rodero B., Alda M., Sobradiel N., Montero J., Moreno S. (2008). Validación De La Versión Española De La Escala De La Catastrofización Ante El Dolor (Pain Catastrophizing Scale) En La Fibromialgia. Med. Clín..

[31] Sullivan M.J.L., Bishop S.R., Pivik J. (1995). The Pain Catastrophizing Scale: Development and validation. Psychol. Assess..

[32] López-Martínez A.E., Esteve-Zarazaga R., Ramírez-Maestre C. (2011). S517 The Spanish Version of the Pain Anxiety Symptoms Scale (Pass-20): Preliminary Data on its Reliability, Validity and Factorial Structure. Eur. J. Pain Suppl..

[33] McCracken L.M., Zayfert C., Gross R.T. (1992). The Pain Anxiety Symptoms Scale: Development and Validation of a Scale to Measure Fear of Pain. Pain.

[34] Bendayan R., Esteve R., Blanca M.J. (2011). New Empirical Evidence of the Validity of the Chronic Pain Acceptance Questionnaire: The Differential Influence of Activity Engagement and Pain Willingness on Adjustment to Chronic Pain. Br. J. Heal. Psychol..

[35] McCracken L.M., Vowles K.E., Eccleston C. (2004). Acceptance of Chronic Pain: Component Analysis and a Revised Assessment Method. Pain.

[36] Ramírez-Maestre C., Esteve R. (2015). A New Version of the Impairment and Functioning Inventory for Patients with Chronic Pain (IFI-R). PM&R.

[37] Little P., Somerville J., Williamson I., Warner G., Moore M., Wiles R., George S., Smith A., Peveler R. (2001). Psychosocial, Lifestyle, and Health Status Variables in Predicting High Attendance among Adults. Br. J. Gen. Prac..

[38] Aban I.B., Cutter G.R., Mavinga N. (2009). Inferences and Power Analysis Concerning Two Negative Binomial Distributions with an Application to MRI Lesion Counts Data. Comput. Stat. Data Anal..

[39] Augustson E.M. (1999). Issues of Acceptance in Chronic Pain Populations. Behav. Anal. Today.

[40] Eccleston C., Williams A.C., Rogers W.S. (1997). Patients’ and Professionals’ Understandings of the Causes of Chronic Pain: Blame, Responsibility and Identity Protection. Soc. Sci. Med..

[41] Thorne F.M., Morley S. (2009). Prospective Judgments of Acceptable Outcomes for Pain, Interference and Activity: Patient-Determined Outcome Criteria. Pain.

[42] Kossakowska K., Szczepanik M., Woszczak M. (2018). Factors of Subjective Assessment of the Effectiveness of Physiotherapy: A Study on Patients with Degenerative Disease of the Spine. Fam. Med. Prim. Care Rev..

[43] Gardner T., Refshauge K., McAuley J.H., Goodall S., Hübscher M., Smith L. (2015). Patient Led Goal Setting in Chronic Low Back Pain—What Goals are Important to the Patient and Are they Aligned to What We Measure?. Patient Educ. Couns..

[44] Jack K., McLean S.M., Moffett J.K., Gardiner E. (2010). Barriers to Treatment Adherence in Physiotherapy Outpatient Clinics: A Systematic Review. Man. Ther..

[45] Peek K., Sanson-Fisher R., Mackenzie L., Carey M. (2016). Interventions to Aid Patient Adherence to Physiotherapist Prescribed Self-Management Strategies: A Systematic Review. Physiotherapy.

[46] Pike A., Hearn L., Williams A.C. (2016). Effectiveness of Psychological Interventions for Chronic Pain on Health Care Use and Work Absence: Systematic Review and Meta-Analysis. Pain.

[47] Gilpin H.R., Keyes A., Stahl D.R., Greig R., McCracken L.M. (2017). Predictors of Treatment Outcome in Contextual Cognitive and Behavioral Therapies for Chronic Pain: A systematic review. J. Pain.

[48] Glenn B., Burns J.W. (2003). Pain Self-Management in the Process and Outcome of Multidisciplinary Treatment of Chronic Pain: Evaluation of a Stage of Change Model. J. Behav. Med..

[49] Jensen M.P., Nielson W.R., Kerns R.D. (2003). Toward the Development of a Motivational Model of Pain Self-Management. J. Pain.

[50] Mun C.J., Otis J.D., Concato J., Reid M.C., Burg M.M., Czlapinski R., Kerns R.D. (2019). Further Examination of the Pain Stages of Change Questionnaires Among Chronic Low Back Pain Patients. Clin. J. Pain.

[51] Qaseem A., Wilt T.J., McLean R.M., Forciea M.A. (2017). Clinical Guidelines Committee of the American College of Physicians Noninvasive Treatments for Acute, Subacute, and Chronic Low Back Pain: A Clinical Practice Guideline From the American College of Physicians. Ann. Intern. Med..

[52] Wideman T.H., Sullivan M.J. (2011). Differential Predictors of the Long-Term Levels of Pain Intensity, Work Disability, Healthcare Use, and Medication Use in a Sample of Workers’ Compensation Claimants. Pain.

[53] Wideman T.H., Asmundson G.G.J., Smeets R.J.E.M., Zautra A.J., Simmonds M.J., Sullivan M.J.L., Haythornthwaite J.A., Edwards R.R. (2013). Rethinking the Fear Avoidance Model: Toward a Multidimensional Framework of Pain-Related Disability. Pain.

[54] Fillingim R.B., Legato M.J. (2017). Sex, Gender, and Pain. Principles of Gender-Specific Medicine: Gender in the Genomic Era.

[55] Samulowitz A., Gremyr I., Eriksson E., Hensing G. (2018). “Brave Men” and “Emotional Women”: A Theory-Guided Literature Review on Gender Bias in Health Care and Gendered Norms towards Patients with Chronic Pain. Pain Res. Manag..

[56] Bhandari A., Wagner T. (2006). Self-Reported Utilization of Health Care Services: Improving Measurement and Accuracy. Med. Care Res. Rev..

[57] Clewley D., Rhon D., Flynn T., Koppenhaver S., Cook C. (2018). Health Seeking Behavior as a Predictor of Healthcare Utilization in a Population of Patients with Spinal Pain. PLoS ONE.

